# Marine Biodiversity in South Africa: An Evaluation of Current States of Knowledge

**DOI:** 10.1371/journal.pone.0012008

**Published:** 2010-08-02

**Authors:** Charles L. Griffiths, Tamara B. Robinson, Louise Lange, Angela Mead

**Affiliations:** 1 Zoology Department, Marine Biology Research Centre, University of Cape Town, Rondebosch, South Africa; 2 Zoology Department, Centre for Invasion Biology, University of Cape Town, Rondebosch, South Africa; Smithsonian's National Zoological Park, United States of America

## Abstract

Continental South Africa has a coastline of some 3,650 km and an Exclusive Economic Zone (EEZ) of just over 1 million km^2^. Waters in the EEZ extend to a depth of 5,700 m, with more than 65% deeper than 2,000 m. Despite its status as a developing nation, South Africa has a relatively strong history of marine taxonomic research and maintains comprehensive and well-curated museum collections totaling over 291,000 records. Over 3 million locality records from more than 23,000 species have been lodged in the regional AfrOBIS (African Ocean Biogeographic Information System) data center (which stores data from a wider African region). A large number of regional guides to the marine fauna and flora are also available and are listed.

The currently recorded marine biota of South Africa numbers at least 12,914 species, although many taxa, particularly those of small body size, remain poorly documented. The coastal zone is relatively well sampled with some 2,500 samples of benthic invertebrate communities have been taken by grab, dredge, or trawl. Almost none of these samples, however, were collected after 1980, and over 99% of existing samples are from depths shallower than 1,000 m—indeed 83% are from less than 100 m. The abyssal zone thus remains almost completely unexplored.

South Africa has a fairly large industrial fishing industry, of which the largest fisheries are the pelagic (pilchard and anchovy) and demersal (hake) sectors, both focused on the west and south coasts. The east coast has fewer, smaller commercial fisheries, but a high coastal population density, resulting in intense exploitation of inshore resources by recreational and subsistence fishers, and this has resulted in the overexploitation of many coastal fish and invertebrate stocks. South Africa has a small aquaculture industry rearing mussels, oysters, prawns, and abalone—the latter two in land-based facilities.

Compared with many other developing countries, South Africa has a well-conserved coastline, 23% of which is under formal protection, however deeper waters are almost entirely excluded from conservation areas. Marine pollution is confined mainly to the densely populated KwaZulu-Natal coast and the urban centers of Cape Town and Port Elizabeth. Over 120 introduced or cryptogenic marine species have been recorded, but most of these are confined to the few harbors and sheltered sites along the coast.

## Introduction

**Figure 1:**
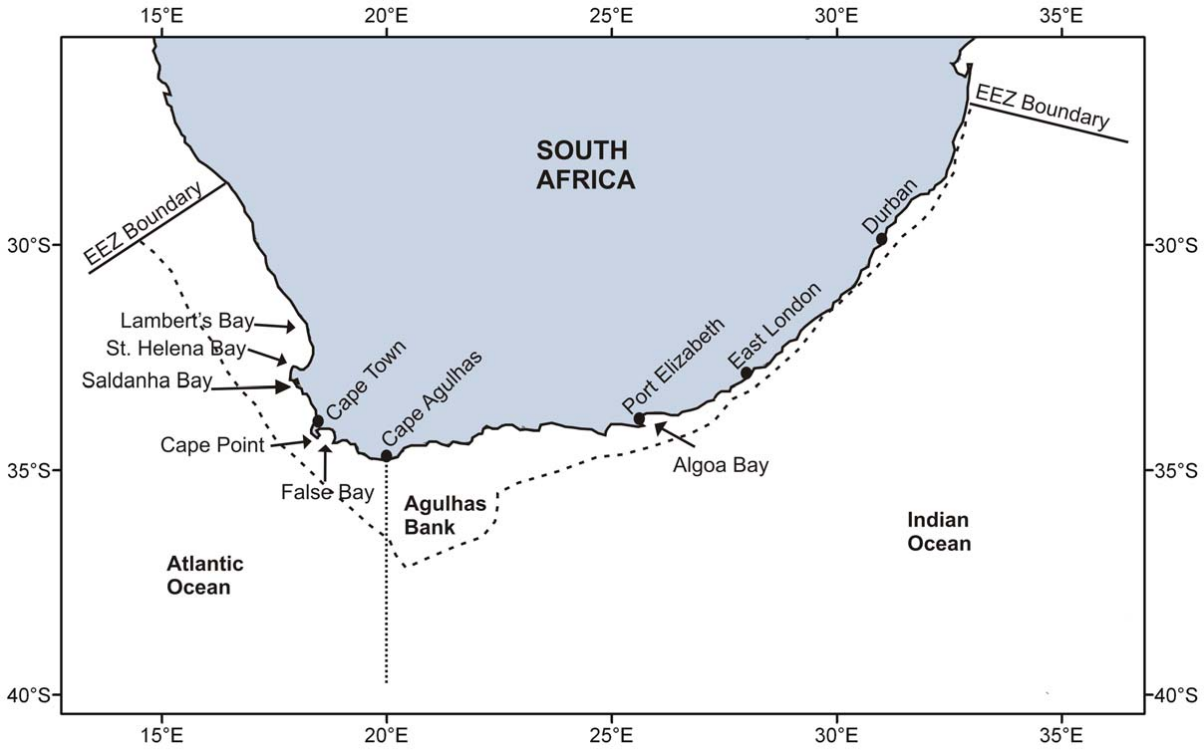
Map of South Africa showing place names mentioned in the text, major current systems, and position of the continental shelf break.

**Figure 2:**
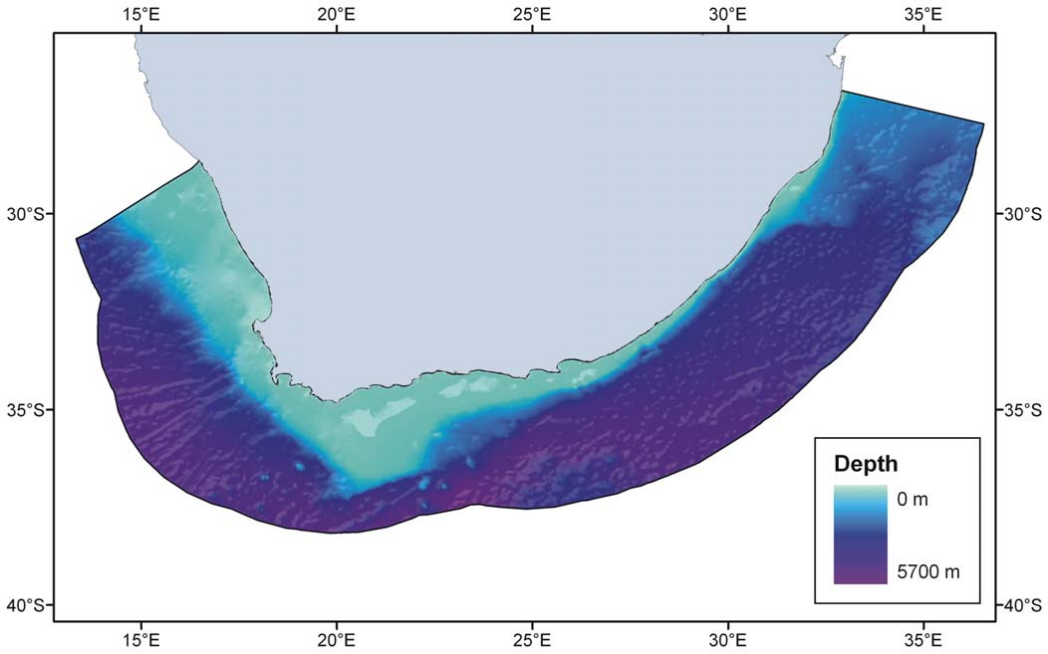
Map showing seafloor depths and the boundaries of South Africa's continental Exclusive Economic Zone (EEZ).

**Figure 3:**
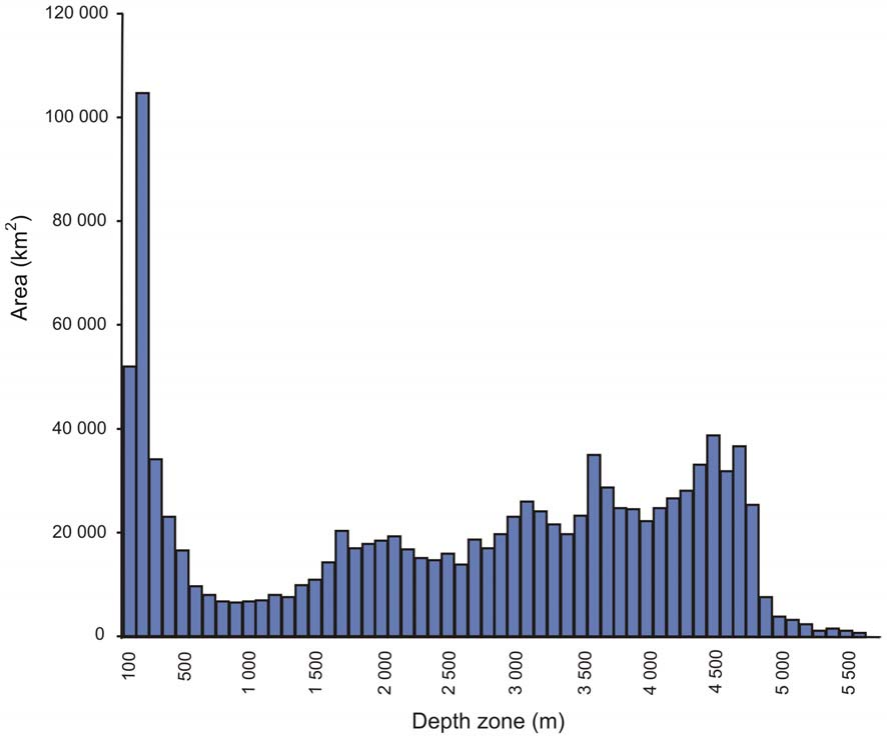
Area occupied by each 100 m depth zone within the South African continental EEZ.

In relation to its land area, South Africa has a short, linear coastline of 3,650 km (Figure 1). The South Africa Exclusive Economic Zone (EEZ) has a total area of 1,535,539 km^2^, of which 466,879 km^2^ surrounds the Prince Edward Islands–South African territories situated in the Southern Ocean and not considered in this analysis. The EEZ surrounding continental South Africa itself (Figure 2) thus has an area of 1,068,659 km^2^, slightly less than the land area of the country, which is 1,221,037 km^2^. The EEZ extends to a maximum depth of 5,700 m and is divided about one-third into the Atlantic Ocean and two-thirds into the Indian Ocean. The continental shelf is narrow along the east (Indian Ocean) coast, but much wider to the west (Atlantic coast) and especially to the south, where it extends into the large, shallow Agulhas Bank. The depth distribution of the South African EEZ is depicted in Figure 3. Only some 25% of the seafloor lies in depths shallower than 1,000 m, with the largest single 100 m depth stratum being 100–200 m, which alone comprises 10% of the entire EEZ. Depths greater than 2,000 m make up 65% of the EEZ, and this region has been subject to extremely little biological sampling (see below).

### Oceanographic regime

The oceanographic regime around South Africa is dominated by two major current systems: the cold Benguela Current along the Atlantic coast to the west and the warm Agulhas Current along the Indian Ocean coast to the east. The Benguela Current has two components. An offshore oceanic flow forms the eastern limb of the South Atlantic Subtropical Gyre and has a broad, sluggish, equatorward flow of only 0.1–0.3 m s^−1^ [1]. Inshore of this a coastal component exhibits dynamic wind-driven upwelling, which is strongly modulated by local weather systems, resulting in short-term upwelling cycles with a periodicity of 5–10 days. Upwelling is concentrated in distinct upwelling cells and occurs predominatly in the austral spring and summer [2]. Offshore, mean monthly sea surface temperatures range from 15.4°C to 20.1°C [3], but in the nearshore upwelling region, variability is greater and temperatures range from 10°C to 18°C [4]. Intense upwelling along the west coast results in high biological productivity, which in turn supports large fish stocks, including pilchard, anchovy, hake, and rock lobster, each forming the basis for lucrative commercial fisheries. Much of the organic matter associated with this high productivity sinks onto the relatively wide continental shelf, where decay results in the reduction of dissolved oxygen in bottom waters [5]. Periodically, these low-oxygen conditions extend close inshore, sometimes reaching the shoreline itself and resulting in mass mortalities of fish, rock lobster, and other invertebrates [6].

Along the east coast, the warm Agulhas Current brings nutrient-poor, tropical waters southward from the equatorial Indian Ocean. The current is strongest and warmest at the shelf break, where surface waters flow at up to 2 m s^−1^ and temperatures vary from 20°C to 28°C, depending on season [7]. Off northern KwaZulu-Natal, the current flows close inshore, but it moves farther offshore as the shelf widens off Durban [8]. South of East London it finally moves well offshore, following the edge of the Agulhas Bank [9] and eventually retroflects south of the country. Intermittently, current reversals result in inshore pockets of cooler water flowing northward, parallel to the coast [7]. These are less predictable farther eastward, but are marked and frequent on the south coast between Cape Agulhas and Port Elizabeth. Close to shore, warm surface layers overlie cool bottom waters during summer [10], but this marked stratification is broken down by winter storms. Periodically, parts of the south coast experience local, wind-driven upwelling of cool bottom water, while the fast flow of the current itself drives upwelling of deep waters, where the shelf widens to form the Agulhas Bank [11]. Productivity on this coast is low and there are few commercial fisheries, although human poulation density is high, resulting in intense pressure on coastal marine resources.

The region between Cape Agulhas and Cape Point is regarded as a region of overlap between south coast and west coast oceanographic regimes. At the point of retroflection of the Agulhas Current, large (∼200–300 km diameter), anticyclonic eddies, termed Agulhas Rings, pinch off into the South Atlantic Ocean [12]. About six such eddies occur per year [13], transporting Indian Ocean water in a northwesterly direction into the Benguela system at 0.05–0.08 m s^−1^ [1].

### The coastline

The South African coastline is 3,650 km in length [14], almost linear in outline, and strongly wave exposed, particularly in the southwest, where peak wave heights exceed 6 m for 10% of the time [15]. There is a simple semidiurnal tidal regime, with spring-tide amplitude 2–2.5 m and neap-tide range about 1 m [16]. Of the few significant bays and inlets on the South African coast, only the Saldanha Bay–Langebaan Lagoon system offers significant shelter along the west coast. Although a number of large, shallow, lunate bays exist on the east coast (e.g., Algoa Bay), False Bay is the only bay along this entire coast deep enough to offer significant shelter from wave exposure. Nonetheless, the many minor rocky headlands offer isolated areas of relative calm, resulting in contrasting wave exposure levels at a local scale [14]. The southern African shoreline consists of approximately 27% rocky shore, 42% sandy beach, and 31% mixed shore—these mostly comprising sand on the upper shore, above a wave-cut rocky platform [17].

There are some 343 estuaries along the South African coast, 292 of which lie along the wetter Indian Ocean coastline. Due to generally low and seasonally variable rainfall, most of these systems are small and seasonally closed. Permanently open estuaries are rare, although the few that do exist support important estuarine habitats [18]. A group of relatively large, shallow saline lakes and lagoons lie along the northern KwaZulu-Natal coast, the largest of which, Lake St. Lucia, covers 300 km^2^ and is the most extensive and best studied estuarine system in the region.

### Biogeography

**Figure 4:**
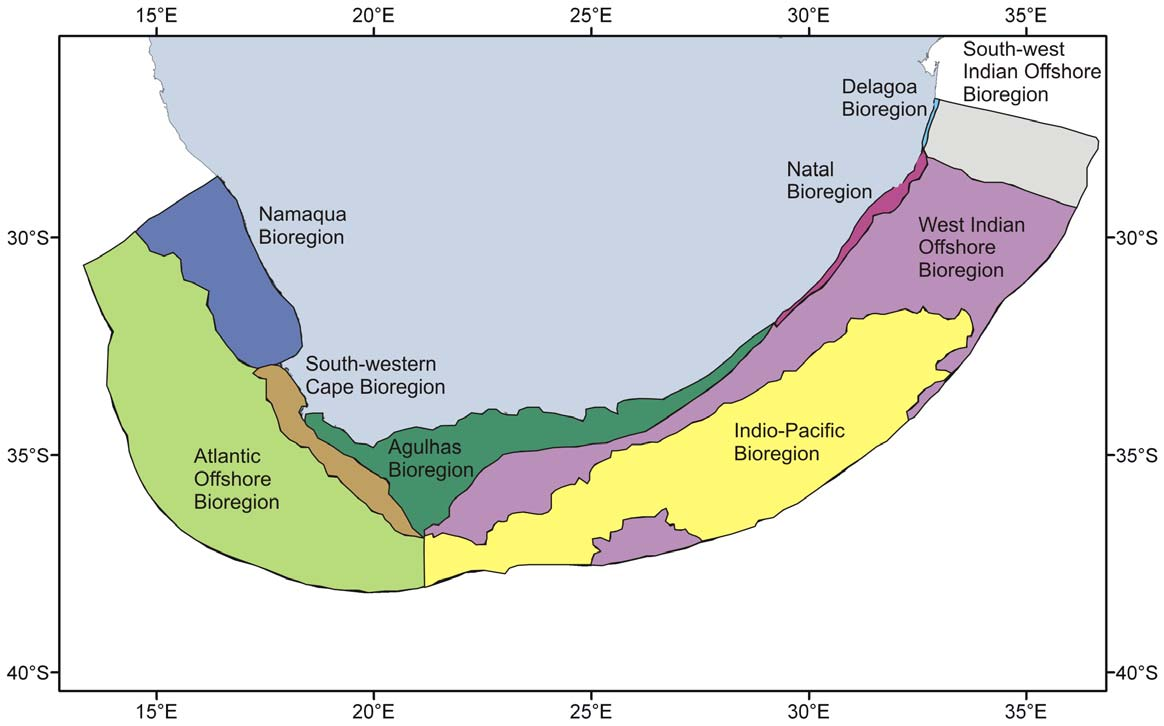
South Africa's nine marine bioregions, as defined by Lombard [**14**].

Many studies have analyzed marine biogeography around the South African coast, and each has recognized between two and five broad coastal biogeographic provinces, with some discrepancies regarding the naming of these areas, levels of dissimilarity between regions, region boundaries, and the recognition of overlap zones [19], [20], [21], [22], [23], [24], [25], [26], [27], [28], [29], [30]. A recent national assessment of marine biodiversity in South Africa has synthesized all existing information and, through extensive expert input, has defined nine marine bioregions, which incorporate both the previously recognized coastal and newly delimited offshore zones, as shown in Figure 4 [14]. Note that while these coastal bioregions have been well defined by means of detailed faunistic and floristic analyses, the offshore regions are defined largely by physical criteria (e.g., temperature, depth, substratum).

In this classification, the coastline is divided into five regions. The cool-temperate Namaqua Bioregion of the west coast and warm-temperate Agulhas Bioregion of the south coast are separated by a broad overlap zone, termed the South-western Cape Bioregion. On the east coast the subtropical Natal Bioregion, merges in the far north of the country into the tropical Delagoa Bioregion, which extends northward into Mozambique. The classification of the offshore environment recognizes four distinct areas. The Atlantic Offshore Bioregion extends from Namibia to Cape Agulhas, while the West Indian Offshore Bioregion includes the continental slopes of the south and east coasts, meeting the tropical South-west Indian Offshore Bioregion in northern KwaZulu-Natal. A deep-water Indio-Pacific Offshore Bioregion includes the abyss of the entire east coast. It must be stressed that within each of these bioregions there certainly exist a variety of localized habitats (e.g., reef, sand, mud), each with their own distinctive biota. Also the boundaries of the offshore bioregions are based on minimal biological sampling and hence may be revised as more quantitative biological field data become available.

### History of exploration

The history of systematic research and exploration in South Africa for virtually all taxa can be divided into three eras [31]. The first, termed the “colonial” phase, was characterized by expeditions that collected specimens from the colonies and shipped them to museums in Europe, where they were cataloged and described, often in beautifully illustrated volumes. The first such dedicated marine collections along South African shores were undertaken in the late 1700s by Carl Peter Thunberg, a student of Carl Linnaeus [32]. These were followed by numerous other collections made by adventurers and naturalists visiting the coasts of Natal and the Cape of Good Hope, and by the great global ocean expeditions, such as the *Challenger*, *Deutschen Tiefsee*, and *Discovery*. The second or “descriptive” phase of research and exploration was dominated by descriptive work, carried out largely, but not exclusively, at South African institutions. In the marine field, this era really began in 1895 with the appointment of J.D.F. Gilchrist as state marine biologist and later as curator at the South African Museum. As a result of his work, and that of his followers, notably the prodigious K.H. Barnard, most common South African marine invertebrate and fish taxa had been fairly well cataloged by the 1970s (see Text S1). From this point, we enter the third or “modern” phase, in which workers began concentrating more on phylogenetic and biological questions and on ecological understanding. A number of important taxa still remain poorly described, and much descriptive work still needs to be done (even within what are regarded as relatively well studied groups). Moreover, our knowledge of the biota of deep-sea environments still remains fragmentary, as will be detailed below.

The most recent development in the field of marine biodiversity has been the Census of Marine Life (Census) program, which has a Sub-Saharan Africa Regional Implementation Committee. This was established in 2003, with the aim of enhancing knowledge about the diversity and distribution of marine life around the African continent—indeed this review is one of the products from that group. The African Census group is supported by a regional data node within the Ocean Biogeographic Information System (OBIS). The AfrOBIS node was set up in 2005 and already holds more than 3.2 million records of more than 23,000 species, the vast majority of these from the seas around Namibia, South Africa, and Mozambique [33]. Only those records from within the political boundaries of South Africa are considered in the more detailed analyses below.

## Methods

### Research capacity

South Africa currently boasts more than a dozen institutions with a strong focus in marine science, and they are fairly well distributed between three main coastal urban centers. The largest concentration of marine scientists is found in the Cape Town region, and includes those based at Marine and Coastal Management (a government directorate within the Departments of Environmental Affairs and of Agriculture, Forestry and Fisheries); the Universities of Cape Town, the Western Cape, and Stellenbosch; the South African (Iziko) Museum; and the Council for Scientific and Industrial Research. A second grouping in the Eastern Cape includes researchers at Nelson Mandela Metropolitan University, Rhodes University, Walter Sizulu University (formerly University of Transkei), and BayWorld (an aquarium and museum complex in Port Elizabeth). The third concentration in the Durban area includes workers at the Oceanographic Research Institute, University of KwaZulu-Natal, Natal Sharks Board, and Ezemvelo KwaZulu-Natal Wildlife. Besides these larger groups, several other institutions, including some distant from the coast, such as the Mammal Research Institute, University of Pretoria, have at least one staff member working in marine science. Despite the apparent good capacity at an institutional level, a different picture emerges at the level of functional ecosystems [34]. While capacity is best in the fields of rocky shore and pelagic open ocean research (particularly with regard to exploited species), expertise on sandy beaches, subtidal hard and soft substrata, and deep-sea environments are generally inadequate. Four large, oceangoing research vessels, all of which are state owned, are currently in use in South African waters. Stock assessments of exploited species are the focus of most research cruises, and little or no ship time is allocated to biodiversity research. In addition, none of the existing vessels possesses the capacity to collect benthic samples from depths deeper than about 1000 m.

As in many other developing countries, taxonomic expertise in South Africa provides only limited coverage, although this greatly exceeds that in any other African country. A list of currently active taxonomists and their fields of expertise is included in Table S1, and a list of major taxonomic reference works and guides to the regional marine biota in Text S1. The coverage of available guides is good, although many of these are now severely dated. A total of 31 local marine taxonomists are active in the region, but many of these are graduate students undertaking taxonomic theses, university staff with a part-time interest in taxonomy, or are retired, but still actively publishing. Only about five of the listed experts are employed as full-time systematists. Current local expertise is also completely lacking for a number of important taxa, particularly those with small body size and little economic significance, such as Hydrozoa, Nematoda, and most Platyhelminthes.

### Marine collections

The primary marine invertebrate collections in the region are housed at the Iziko South African Museum in Cape Town and comprise some 129,000 records, offering significant coverage of all major marine taxonomic groups. Other, more specialized collections are housed at several other museums spread around the coast, notably the national fish collection at the South African Institute for Aquatic Biodiversity in Grahamstown (56,000 records) and the collection of mollusks at the Natal Museum (63,000 records). Large collections of algae are also held by the Bolus Herbarium at the University of Cape Town (11,000 records) and the Schoenland Herbarium at Rhodes University (32,000 records).

### Sample coverage

Sampling effort has been best for intertidal habitats, where there is good coverage around the whole South African coast, undertaken primarily by the University of Cape Town Ecological Survey, allowing for detailed mapping of habitat types [35] and species distributions [36] on a national scale. Shallow nearshore waters have also received relatively good attention, allowing for detailed analyses of the distribution patterns of coastal fish [28], various invertebrate groups [37], and algae [38], among others.

**Figure 5:**
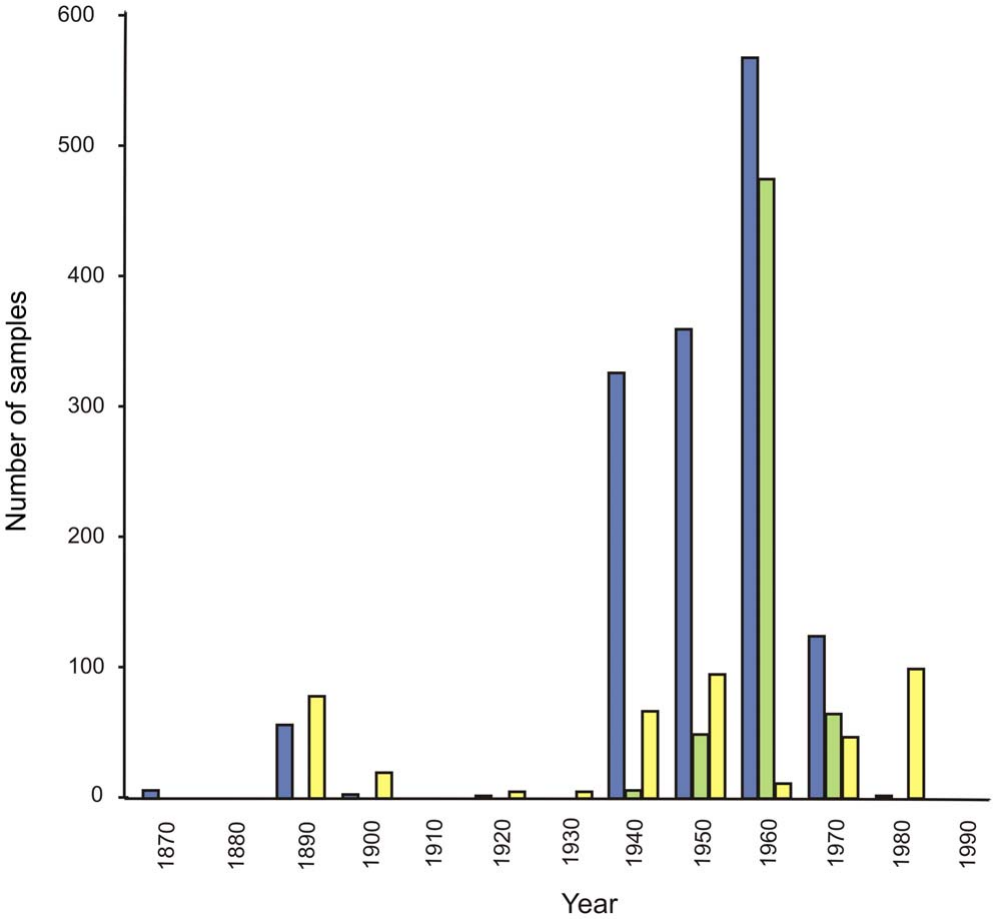
Number of benthic invertebrate samples collected within South African waters each decade. Samples coded by method: dredges (blue), grabs (green), and trawls (yellow).

**Figure 6:**
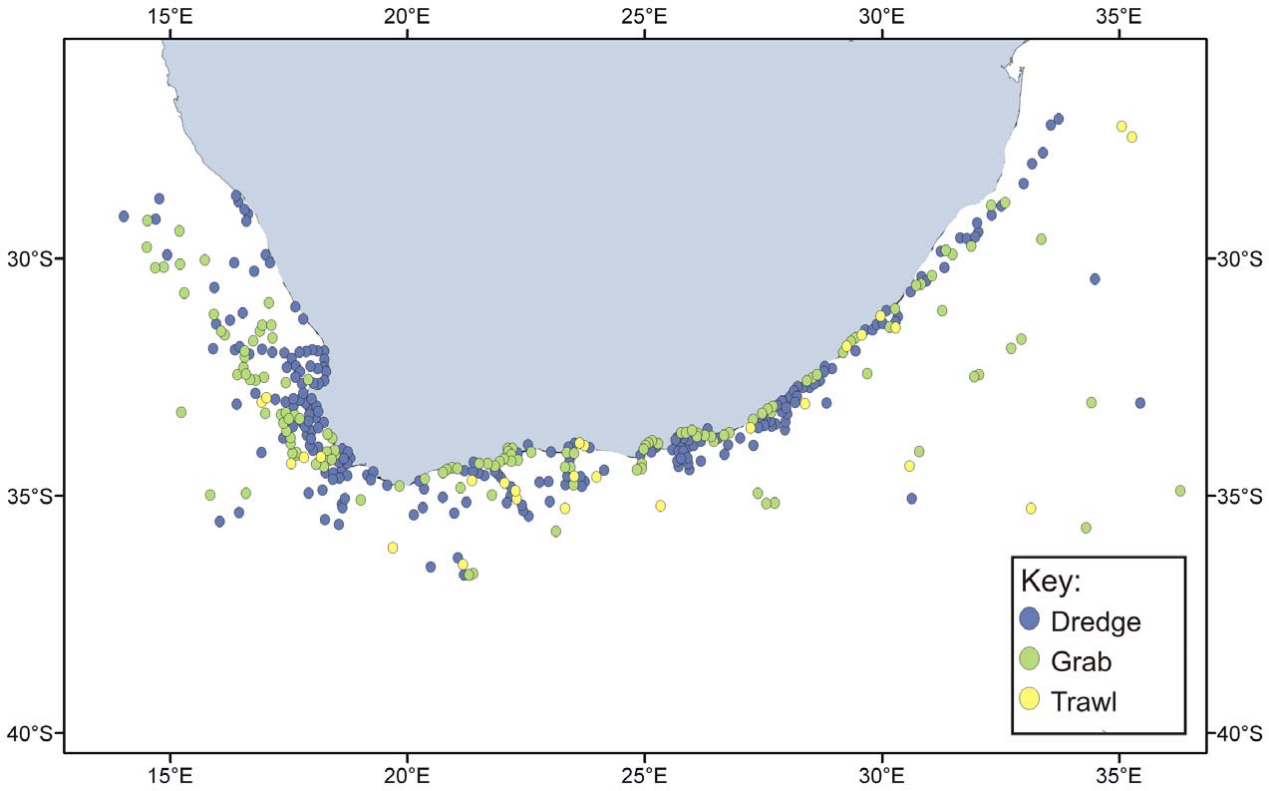
Locations of benthic invertebrate samples collected by dredges (blue), grabs (green), and trawls (yellow) around the South African coast.

**Figure 7:**
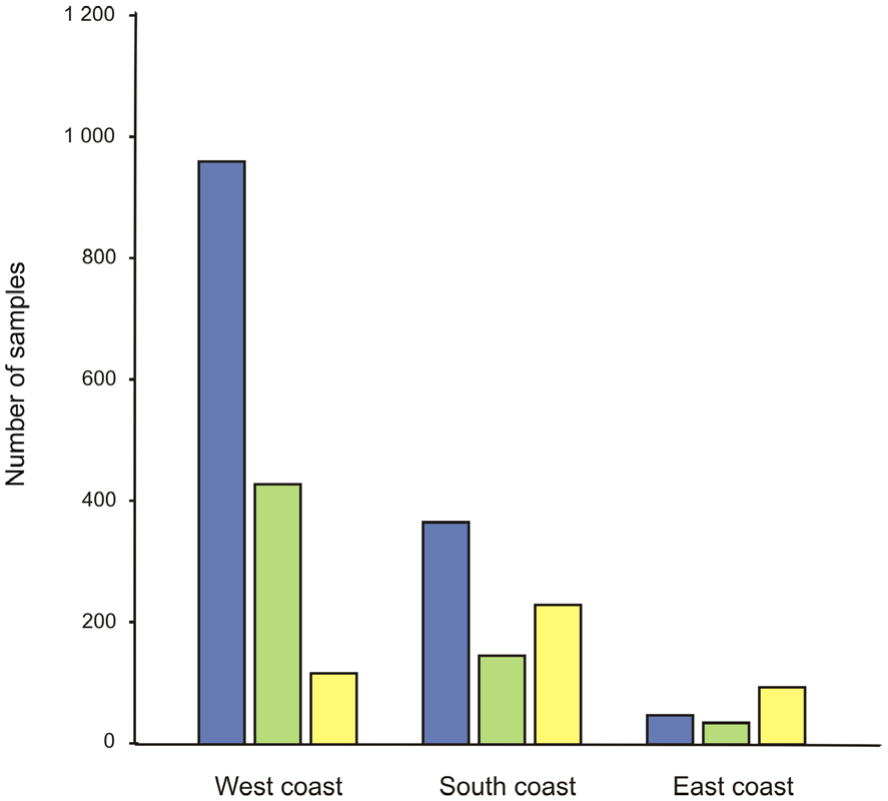
Regional distribution of benthic invertebrate samples collected in South African waters. Dredge, grab, and trawl samples are represented in blue, green, and yellow, respectively.

By contrast, biodiversity over the greater part of the offshore continental shelf around South Africa is less well documented [39]. An exception is the ichthyofauna, which has been well studied, largely as a result of regular stock assessment surveys undertaken in support of the region's major demersal, pelagic, and line-fish fisheries. Current knowledge of benthic invertebrate diversity and biogeography is based on some 1,460 dredge, 602 grab, and 442 trawl samples, which have been analyzed for community structure. Many more samples exist in museum collections, but the majority of these samples originate from directed collections of individual species or taxa, rather than collections that examine the composition of the entire community. Some of the early samples originate from international expeditions of the late 1800s and early 1900s, such as the *Challenger*, *Valdivia*, and *Gauss*, but the vast majority of samples were collected during the University of Cape Town Ecological Survey, which took place from the 1940s to early 1980s. Virtually no benthic invertebrate surveys have been undertaken since that time, as shown in a plot of the temporal sequence of sample collection (Figure 5). The majority of benthic samples are from the west coast (Figure 6), where several inshore sites have been particularly well sampled, notably Lambert's Bay, St. Helena Bay, Saldanha Bay/Langebaan Lagoon, Table Bay, and False Bay. The south coast shelf is also moderately well sampled, while KwaZulu-Natal has by far the least number of samples. Most of the samples on both the west and south coasts were collected by dredging, while trawling was the dominant collection method utilized off the east coast (Figure 7).

**Figure 8:**
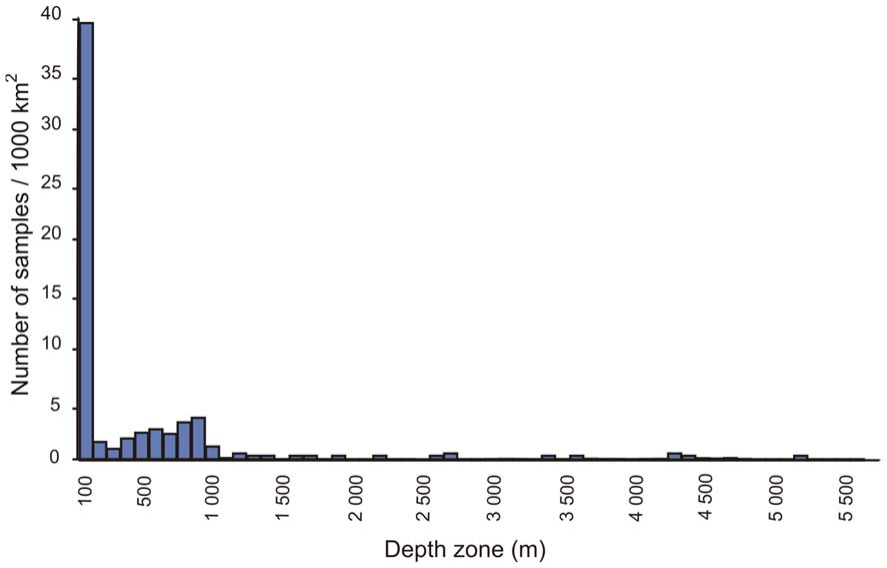
Number of benthic samples taken per 1,000 km^2^ in each 100 m depth zone within the South African EEZ.

The depth distribution of all existing benthic samples shows that the bulk of these (83%) have been taken in less than 100 m of water, while only 2% have been taken in water deeper than 1,000 m. Comparison of the numbers of samples with the area per depth zone (Figure 8) reveals that 39 samples have been taken per 1,000 km^2^ in the 0–100 m depth zone (by far the most in any zone). At depths of 100 to 1,000 m, between one and five samples have been taken per 1,000 km^2^, while deeper than 1,000 m all depth zones have less than one sample per 1,000 km^2^ and most strata remain totally unsampled! This lack of data severely constrains the assessment of patterns of benthic biodiversity in South African waters. Our knowledge of the biota is further complicated by the fact that many macrofaunal species still remain to be formally described.

## Results

### Known biodiversity

South Africa is widely recognized as a region of high biological diversity, and in terrestrial species it is the third most diverse country in the world [40]. The species richness of South African marine systems, however, has rarely been placed in a global context, although the reviews in this collection will assist in providing just such an analysis.

Table 1 provides a summary of the numbers of known marine species in South Africa by major taxa and evaluates the state of knowledge of each, while Table S1 gives the same information in much more detail, broken down by class or order. The currently known number of marine species from South Africa is estimated at 12,914. Of course, this number constantly changes, as species are described as new to science, or are newly recorded from the region, or as existing species are subjected to taxonomic revision. The species richness reported here is a considerable increase from the 11,130 faunal species given in a previous (1999) synthesis [41]. There are several reasons for this increase. Additional taxa have been described or newly recorded in the region over the past decade, primarily among the Porifera [42], Bryozoa [43], and Tunicata [44]. We have also been able to trace some records that were not included by Gibbons et al. [40], such as additions to the Ciliophora, Dinozoa, Myxozoa, Phoronida, Platyhelminthes, and Rotifera. However, the most important factor adding to the current estimate of species diversity is that our listing includes algae and fungi, which were not considered in the previous compilation [41]. By far the most speciose taxa listed are the Mollusca (3,154 species), Arthropoda (2,451 species), and Pisces (2,000 species), which are the only groups containing more than 1,000 species each, and which together account for no less than 68% of the total biota in South African waters.

**Table 1:** Summary of known marine biodiversity in South Africa.

Taxonomic group	No. species^1^	State of knowledge	No. introduced spp.	No. experts	No. ID guides^2^
**Domain Archaea**	n/a	1	n/a	0	0
**Domain Bacteria (including Cyanobacteria)**	n/a	1	n/a	0	0
**Fungi**	1	1	0	0	0
**Domain Eukarya**					
**Kingdom Chromista**					
Phaeophyta	111	5	0	2	2
Other Chromista	225	2	n/a	2	3
**Kingdom Plantae**					
Chlorophyta	197	5	1	4	2
Rhodophyta	505	5	3	4	2
Angiospermae	7	5	2	0	4
**Kingdom Protoctista (Protozoa)**					
Dinomastigota (Dinoflagellata)	220	3	3	1	0
Foraminifera	15	2	0	1	0
**Kingdom Animalia**					
Porifera	346	3	1	1	1
Cnidaria	853	3	13	4	9
Platyhelminthes	354	2	0	1	0
Mollusca	3154	4	11	1	10
Annelida	787	3	7	1	1
Crustacea	2331	3	21	4	9
Bryozoa	270	3	6	1	1
Echinodermata	410	4	2	2	5
Urochordata (Tunicata etc)	227	3	9	1	3
Other invertebrates	630	3	3	2	8
Vertebrata (Pisces)	2000	5	1	5	6
Other vertebrates	272	5	0	1	7
**SUB-TOTAL**					
**TOTAL REGIONAL DIVERSITY** 3	12915				

**Notes:**

1 Sources of the reports: databases, scientific literature, books, field guides, technical reports.

2 State of knowledge is ranked on a scale of 1–5, where 1 = very poor or unknown and 5 = well known, n/a = no data available. For a more detailed breakdown by class and order, see Table S1.

3 Number of introduced species follows Mead et al. (in review) and excludes cryptogenic species.

4 Identification guides lists major works only, as cited in Text S1.

5 Total regional diversity including all taxonomic groups as reported in Table S1.

We recognize that the quality of the data in Table S1 is mixed. Even at the phylum level, no species are reported from eight phyla, while only a single species is known from a further three (Rotifera, Kinorhyncha, Fungi). It is very likely that this reflects lack of taxonomic attention, rather than actual absence or paucity of these groups from the region. Current species counts within many other groups, particularly of smaller organisms, such as Platyhelminthes, Nematodes, Chaetognatha, and Protocista, are also likely to be greatly underestimated. We have attempted to estimate the number of unidentified species in each group using a method described by Griffiths [45]. This technique involves comparing the ratio of species in well-studied groups between South Africa and Europe (the best-studied region in the world) and then assuming that a similar ratio should apply to those groups that have been poorly studied in South Africa. Using this approximation, we estimate that 7,590 additional species need to be described to bring the state of taxonomic knowledge in South Africa up to European levels (Table S1). It should be noted that for all groups for which the present number of known species was not available, no estimate of unknown species could be calculated, and that this results in an underestimation of the total number of species from the region. Additionally, this overall estimate remains a minimal one of absolute biodiversity, since even in European seas species continue to be discovered at a rate that has remained linear for the past 300 years, being limited more by the availability of taxonomic expertise, than of new material to describe!

Not surprisingly, higher vertebrates are considered to be well-documented, and where no species have been recorded (Crocodylia and Sauria) it is with confidence that we report that none are present and it is only within the Pisces that new vertebrate species continue to be regularly discovered. Other taxa that are relatively well documented include the Echinodermata, several groups within the Crustacean (Amphipoda, Isopoda, Decapoda), Polychaeta, Mollusca, Bryozoa, Cnidaria, and the macroalgae (Rhodophyta, Chlorophyta, and Phaeophyta). Major groups that are still considered to be greatly underestimated include Tunicata, Platyhelminthes and Nematoda.

The total number of endemic species listed in Table S1 is 4,233, or 33% of the listed biota. Such estimates of endemism are subject to error, both because the number of endemic species is simply not available for some groups, and because poor levels of taxonomic research in adjoining countries (as is the case here) tend to artificially elevate apparent rates of endemism. Moreover, these (and most earlier) estimates are derived from published literature, and some species listed may have subsequently ceased to be endemic because they have since been recorded in another country. Nevertheless, available data suggest that South Africa supports a high proportion of marine endemic species, although this is highly variable among taxa. At the level of major phyla, Bryozoa and Mollusca demonstrate very high levels of endemism—64% and 56%, respectively—in contrast to much lower proportions shown by phyla such as Echinodermata (3.6%) and Porifera (8.8%). Marked differences are also evident even between closely-related groups, including those with similar life histories. For example, the proportion of endemics among the Amphipoda is 33%, far lower than that among the Isopoda (85%), or Cumacea (71%). These differences are hard to explain, but may arise from differences in the state of research in adjoining counties (poorer reporting in adjoining countries tending to increase apparent rates of endemism).

### Spatial patterns

**Figure 9:**
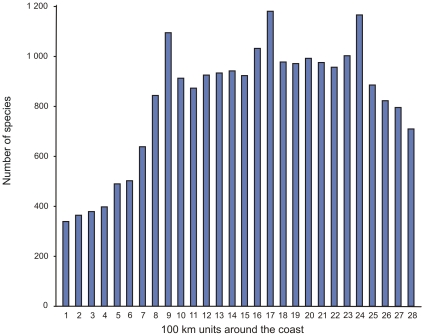
Number of species recorded from each 100 km unit around the coast of South Africa from the Namibian border (1) to the Mozambique border (28). Cape Town is in unit 9 and Durban in unit 24.

The spatial patterns of species richness and endemism of coastal fishes, macroalgae, and a variety of benthic invertebrate groups around the South African coast have been plotted [24], [28], [30], [37]. The main findings of these studies were that some groups, including fishes, bivalves, gastropods, brachyurans, and echinoderms, become progressively more species rich to the (more tropical) east, whereas other taxa, such as amphipods, isopods, and polychaetes, attain maximum species richness in the temperate southwest. When all groups are summed, the pattern is one of low species richness along the entire west coast and relatively even species richness along the remainder of the coast (Figure 9). The apparent decline in species to the extreme east is almost certainly due to reduced sampling intensity in that region (see above). Endemicity in all groups peaks along the south coast, but to a large extent this may be an artifact of the way endemism is defined (as being confined within the political borders of a single country)—since the proportions of endemics naturally tends to increase with linear distance from the nearest political border.

Another way of examining these data is to plot the distribution patterns of range-restricted species, such as those with ranges of 300 km or less. Interestingly, the resulting plot (37) shows that range-restricted species are strongly concentrated on the boundaries or “ecotones” where two biogeographic regions meet, particularly around Cape Point.

### Introduced species

The most recent published account of marine alien species in South Africa [46] lists only 22 confirmed alien species and 18 cryptogenic species. However, unpublished work by the authors has raised these numbers to 86 introduced species and an additional 40 cryptogenic species (Mead *et al.* in prep), with more newly discovered introductions regularly being added to this list. Most of these introduced species are confined to sheltered sites, such as harbors, lagoons and estuaries and only two—the Mediterranean mussel (*Mytilus galloprovincialis*) and the Pacific barnacle (*Balanus glandula*)—have become widespread on the open wave-exposed coastline. Two other species—the sponge *Suberites tylobtusa* and the anemone *Metridium senile*—have established significant populations in deeper waters and the impacts of these new populations are currently under investigation. Taxa with the largest numbers of introduced species are the Crustacea (33 species), Mollusca (22 species), Ascidiacea (18 species), and Cnidaria (16 species). The only fish listed is the carp *Cyprinus carpio*, which extends into the upper reaches of estuaries, but not into the sea itself. The low number of alien species recorded from the Protocista, and absence of recorded introductions from groups such as the Fungi, Chromista, Procaryotes, and Bacteria is considered indicative of the poor level of taxonomic knowledge for these groups, rather than any lack of actual introductions.

## Discussion

### The known, unknown, and unknowable

Compared with other developing countries, South Africa has a fairly strong history of taxonomic research and, as a result, the marine fauna of the region is relatively well known, certainly far better so than that of any other African nation. In addition, a comprehensive series of regional identification guides are available dating from the 1950s to the 2000s (Text S1), although many of these are now in urgent need of revision.

Inevitably, given the limited number of active taxonomists in the region, certain taxa (for example, fish, mollusks, crustaceans, polychaetes) have received far more attention than others. Indeed some have been completely neglected (see Table S1). In addition, sampling effort has been strongly biased toward coastal and shallow waters, as vanishingly few benthic samples have been collected in waters deeper than 1,000 m (Figure 8). As a result, there are definite taxonomic, regional, and habitat biases in our current state of knowledge. The most obvious of these is the lack of samples from the abyssal zone (>3,000 m), even though this zone makes up half of the national EEZ. This is largely a consequence of the great cost of collecting such samples, as well as the lack of locally-based capacity to undertake sampling at great depth. Other gaps include sampling of hard substrata in all depths deeper than those accessible to scuba divers (>30 m) and a relative undersampling of the more tropical waters of the north-east coast (Figure 6).

### Value, use, and impacts of biodiversity

The South African marine biota supports a wide range of fisheries (Table 2) that together contribute roughly 1% to the national GDP (approximately US$404 million) [47]. The most valuable fishery in commercial terms is the demersal fishery, which is focused mainly on Cape hake, with additional catches of Agulhas sole, kingklip, and adult horse mackerel, constituting a total nominal catch of 188,842 t [48]. The pelagic fishery for anchovy and pilchards is South Africa's largest fishery in terms of tonnage, with a highly variable annual catch, currently of roughly 600,000 t [47]. The line fishery is the third most important fishery in South Africa in total tonnage landed and total economic value. Although records for the commercial line-fish sector are maintained, landings by the open-access recreational line fishery are not reported, even though the total catch from this sector may be double that reported by the commercial sector [49]. The west coast lobster fishery is one of the oldest along the South African coast, dating back to at least 1875. Commercial, subsistence, and recreational sectors all form part of this fishery. In the last decade substantial stocks have developed along the south coast (an area not traditionally considered commercially viable for rock lobster fishing). This has resulted in a small-scale commercial fishery being opened in this region in 2003 [50].

**Table 2:** Major fisheries sectors in South African waters and the annual catches of each (data derived from references 47, 48, 53).

Fisheries sector	Method	Target species	Annual catch	Region
Demersal fisheries	Trawl	Deep water hake (*Merluccius paradoxus*), Shallow water hake (*Merluccius capensis*)	158,000 t	Deep water west and south coast
		*M. capensis*	±6% of hake TAC	Shallower than 110 m on the Agulhas Bank
		Agulhas sole (*Austroglossus pectoralis*)	872 t	Agulhas Bank, west coast
	Longline	Kingklip (*Genypterus capensis*)	Figure not available	West and south coast
		*M. paradoxus*, *M. capensis*	10% of hake TAC	
	Midwater trawl	Adult horse mackerel (*Trachurus trachurus capensis*)	58,000 t	West and south coast
Pelagic fisheries	Purse-seine	Anchovy (*Engraulis encrasicolus*), Pilchard (*Sardinops sagax*)	600,000 t (both species)	Inshore on west and south coast
		Juvenile horse mackerel and lanternfish	Variable (up to 25,000 t)	Inshore, west and south coast
		Round herring (*Engraulis whiteheadi*)	Infrequent and highly variable	Further offshore than anchovy and pilchards
Line fisheries	Poling	Albacore tuna (*Thunnus alalunga*), yellowfin tuna (*Thunnus albacares*)	4,000–6,000 t	Offshore west coast
	Rod, reel, or handline line fishery	Commercial: Hake, tuna, shark, sword fish and a variety of other species	±18,000 t	The whole coast
		Recreational: A variety of species	3,000 t	Around the whole coast
Beach seine	Seine nets from the beach	Harders (*Liza richardsonii*)	±6,000 t	West and south coast
Chokka squid fishery	Jigging	Chokka squid (*Loligo vulgaris*)	±6,000 t Based on average 1993–2002	South coast
Lobster	Traps set on longlines	South coast spiny lobster (*Palinurus gilchristi*)	382 t (tail mass)	Offshore south coast
	Traps, hoopnets, and recreational divers	West coast rock lobster (*Jasus lalandi*)	3,527 t	West and south coast
Prawn	Trawl	Six shallow water penaeid prawn species	Variable ±100 t	East coast
Wild oysters	Collection from the open coast (commercial and recreational)	Cape rock oyster (*Striostrea margaritacea,*)	Circa 500,000 individuals	East and south
		*Striostrea cuccullata*	Not available	East coast
Abalone	Diving using the “hookah” system	Abalone (*Haliotis midae*)	Fishery collapsed and was officially closed in 2008	
Algal fisheries	Beach cast collected	*Gracilaria verrucosa*	Not available	West coast
	Beach cast collected, live kelp harvested from the shore	Kelp (*Laminaria pallida, Ecklonia maxima*)	7,000 t frond weight	West coast and south coast
	Beach cast collected	*Gelidium* species	Not available	West coast and south coast

Note: Data derived from reference 47.

Seaweeds have been commercially collected since the 1940s for extraction of alginates and agars used as thickeners, gelling agents, stabilizers, and emulsifiers in paints, food, and cosmetics. This industry is thought to have little impact on biodiversity, as plants are collected once they have washed ashore, or are harvested at low intensity by ecologically sustainable methods [51], [52]. Nonetheless, there has been a recent rapid increase in the collection of live kelp as feed for an expanding cultured abalone industry. This has raised concerns regarding future demands that may be placed on kelp resources along some sections of the coast.

While most commercial fisheries are focused on the west and south coasts, the east coast has few and smaller fisheries, but a high coastal population density, resulting in intense exploitation of inshore resources by recreational and subsistence sectors. As a result, many coastal fish and invertebrate stocks in this region are overexploited [53]. The country has a small aquaculture industry rearing some 6,000 t of mussels, oysters, prawns, and abalone—the latter two in land-based facilities. Although the tonnage of abalone produced is moderate, value is high and South Africa ranks as the third-largest global producer of this product.

In the last decade, ecotourism based on South Africa's marine environment has developed significantly. In particular, shark, whale, and dolphin watching have rapidly expanded. Along the south coast, a thriving industry exists round boat-based viewing and cage diving with great white sharks (*Carcharodon carcharias*), while a number of shark species and large pods of dolphin attract tourists along the KwaZulu-Natal coast. Besides these directed industries, tourists (both South African and foreign) make extensive use of the South African coast for recreational purposes.

### Threats to biodiversity

South African marine biodiversity is under threat from a range of anthropogenic activities, the intensity and variety of which have increased significantly over the past hundred years. With reference to the coastal zone, impacts include direct exploitation, the introduction of non-native marine species, climate change, habitat modification, pollution, and disturbance.

Direct exploitation of coastal resources ranges from traditional subsistence exploitation and recreational fishing to full-scale commercial activities. Following global trends, overall landings by South African fisheries increased dramatically from the 1950s [53], [54], [55], but subsequently declined from an unsustainable peak and are now relatively stable. Details of the various fisheries sectors are provided in the previous section.

Coastal impacts of climate change include rise in sea level and changes in circulatory and sea surface temperature patterns. Increasing sea level is not predicted to be of great consequence to most coastal species, as they can simply move higher up on the shore. An exception might occur on the South African east coast, where many shores consist of rock platforms in the lower shore, bounded by sandy habitats above. Here rising sea levels may result in the loss of habitat for some upper intertidal species. Of more importance are changes in the geographic ranges of species associated with changing sea temperature. Along the east coast rising sea temperatures can be expected to result in the southward expansion of the ranges of tropical species. Unexpectedly, though, recent satellite evidence suggests that between 1987 and 2007, temperatures have in fact declined along the west and south coasts (Rouault, personal communication). This decline is due to shifts in wind and rainfall patterns, resulting in changes in upwelling patterns, a well-known effect of climate change [56]. One example of a significant climate-induced change in community composition has been detected in False Bay. This location falls in the transition zone between cold west coast and warm-temperate south coast conditions and has seen declines in the warm-water indigenous brown mussel (*Perna perna*) and concurrent increases in kelp and the cold-water invasive mussel (*M. galloprovincialis*) (Mead and Griffiths, in review). A strong movement in the center of gravity of both pelagic fish and West Coast rock lobster [57] stocks from west to east over the past decade, presumably initiated by climate change, has also taken place. The resulting change in availability of prey has in turn caused dramatic declines in the numbers of predatory seabirds in west coast colonies and corresponding increases in the size of those colonies on the south and east coast over the same period [58].

Harbors, marinas, seawalls, railway lines, and other structures constructed along the seashore are common features in South Africa's coastal cities [55]. Although these forms of development are spatially limited, they will have displaced organisms. Similarly, near- and offshore pipelines continue to discharge increasing volumes of sewage, fish waste, or industrial effluent into the marine environment. The nearshore pipelines are concentrated around a few major population centers, leaving most of the coast unaffected, but a formal national assessment of the impact of these releases has not been made. Disturbance due to human trampling and diving activities is thought to be limited, both spatially and temporally, being focused around relatively few key recreational areas and during holiday periods. While South Africa's progressive environmental legislation prohibits the use of motor vehicles in sensitive beach zones and controls the approaching of marine mammals, recent work has demonstrated that food provisioning by the shark-watching industry has a negligible impact on shark behavior [59].

Currently, 23% of the South African coastline, but less than 1% of the country's EEZ, falls within marine protected areas (MPAs) [14]. Although the proportion of coastline in declared MPAs is high, there is concern that only 9% of coastal protected areas enjoy total protection (no-take MPAs). In addition, existing MPAs are unevenly distributed among the five coastal bioregions. The entire Namaqua Bioregion currently lacks any MPA, although the proposed proclamation of a Namaqualand MPA, extending from the coast to include offshore habitats, would increase the area under protection. By contrast, the Delagoa Bioregion, on the east coast, receives 20% protection in no-take MPAs [14]. This spatial imbalance results in a large portion of South Africa's coastal marine biodiversity remaining unprotected. The conservation status of offshore regions is of even greater concern, as less than 0.2% receives total protection [14]. It is important to note, however, that without adequate enforcement, MPAs do little to conserve the organisms and habitats within their boundaries. The capacity for such enforcement, even in South Africa's present MPAs, is questionable and cause for concern.

While the protection of biodiversity in general is clearly a key aim and achievement of these MPAs, the adequate protection of specific taxa (such as the intensively illegally harvested abalone) and specific key habitats may still require additional dedicated efforts. A recent spatial assessment of South African marine biodiversity [14] noted the fish fauna as the most exploited and threatened major component of the marine biota, while high-profile reefs and pinnacles, soft-bottom trawling grounds, and coastal and subtidal areas exposed to mining on the west coast were identified as the most threatened habitats. Through the establishment of an accurate fish distribution database and detailed mapping and sampling of the habitats named above, future research could significantly enhance the level of protection afforded to South African marine biodiversity.

On a final positive note, there is enormous scope for future marine biodiversity research in South Africa. The large numbers of undescribed species in a variety of taxa are indicative of the wide potential for future species discovery. A new generation of taxonomists will be needed to perform these tasks, but the recent creation of a South African National Biodiversity Institute (SANBI) and South African Biosystematics Initiative (SABI) has increased the availability of funding and encouraged young researchers to enter this field. Other key areas that require attention include quantification of the effect of trawling and mining on benthic habitats, assessment of the impacts of alien species, quantification of the impacts of pollution (sewage and storm water) in the nearshore environment, and the quantification and prediction of future climate change effects.

## Supporting Information

Text S1 Major taxonomic resources and guides to the South African marine biota.
(0.08 MB DOC)

Table S1 (0.04 MB XLS)
