# Supplementary material for: Marine Biodiversity in South Africa: An Evaluation of Current States of Knowledge
Source: PLoS One. 2010 Aug 2;5(8):e12008. doi: 10.1371/journal.pone.0012008 (PMC2914023; doi:10.1371/journal.pone.0012008)
Supplement: Text S1 — Major taxonomic resources and guides to the South African marine biota. (0.08 MB DOC) [file pone.0012008.s001.doc]

# Text S1: Major taxonomic resources and guides to the South African marine biota.

## General works

Branch M (1987) Explore the seashore of South Africa. Cape Town: Struik Publishers. 48 p.

Branch G, Branch M (1981) The living shores of southern Africa. Cape Town: Struik Publishers. 272 p.

Branch G, Griffiths CL, Branch M, Beckley LE (1994) Two oceans: A guide to the marine life of southern Africa. Cape Town: David Philip Publishers. 360 p.

Day JH (1974) A guide to marine life on South African shores. Cape Town: A.A. Balkema. 272 p.

Griffiths CL, Griffiths R, Thorpe D (1988) Seashore life. Struik Pocket Guide Series, Cape Town: Struik Publishers. 64 p.

Kalk M (ed.) (1995) A natural history of Inhaca Island, Mozambique (3rd ed). Johannesburg: Witwatersrand University Press. 395 p.

Lubke R, De Moore I (eds.) (1998) Field guide to the Eastern and Southern Cape coasts. University of Cape Town Press in association with Grahamstown Branch, Wildlife and Environment Society of South Africa. 559 p.

Payne AIL, Crawford RJM, Van Dalsen A (1989) Oceans of life off southern Africa. Cape Town: Vlaeberg Publishers. 380 p.

Richmond MD (ed.) (2002) A guide to the seashores of eastern Africa and the western Indian Ocean islands (2nd ed). SIDA/SAREC-UDSM. 461 p.

Wirtz P, Debelius H (2003) Mediterranean and Atlantic invertebrate guide. Hackenheim: Conchbooks. 305 p.

## Identification guides to specific groups

### Mammalia and Reptilia

Best PB (2007) Whales and dolphins of the southern Africa subregion. Oxford: Cambridge University Press. 338 p.

Branch WR, Ross GJR (1988) Marine reptiles and mammals. In: A field guide to the Eastern Cape coast. Lubke R, Gess F, Bruton M, editors. Grahamstown Centre of the Wildlife Society of Southern Africa. pp. 115–130.

Skinner JD, Smithers RHN (1990) The mammals of the southern African subregion. Pretoria: University of Pretoria. 771 p.

### Aves

Ginn PJ, McIlleron WG, Milstein P, le S (1989) The complete bird book of southern Africa. Cape Town: Struik Winchester. 760 p.

Maclean GL (1993). Robert's birds of southern Africa (6^th^ d). Cape Town: JohnVoelcker Bird Book Fund. 871 p.

Newman K (2002) Newman's birds of southern Africa (8th ed). London: Struik. 512 p.

Sinclair JC, Hockey PAR, Tarboton WR (1998). Sasol birds of southern Africa (3^rd^ ed.) Cape Town: Struik. 447 p.

### Pisces

Compagno LJV, Ebert DA, Smale MJ (1989) Guide to the sharks and rays of southern Africa. Cape Town: Struik Publishers. 160 p.

Heemstra P, Heemstra E (2003) Coastal fishes of South Africa. Grahamstown: NISC and SAIAB. 488 p.

King D (1996) Reef fishes and corals: East coast of southern Africa. Cape Town: Struik Publishers. 128 p.

King D, Fraser V (2002) More reef fishes and nudibranchs. Cape Town Struik Publishers. 136 p.

Van Der Elst R (1988) A guide to the common sea fishes of southern Africa (2nd ed). Cape Town Struik Publishers. 398 p.

Van Der Elst R (1990) Everyone's guide to sea fishes of southern Africa. Cape Town Struik Publishers. 112 p.

### Ascidiacea

Millar RH (1955) On a collection of ascidians from South Africa. Proc. Zool Soc. Loud. 125: 169–221.

Millar RH (1962) Further descriptions of South African ascidians. Ann. S. Ajr. Mus. 46: 113–221.

Monniot C, Monniot F, Griffiths CL, Schleyer M (2001) South African ascidians. Ann. S. Afr. Mus. 108: 1–141.

### Echinodermata

Balinsky JB (1957) The Ophiuroidea of Inhaca Island. Ann. Natal Mus. 14: 1–33

Clark AM, Courtman-Stock J (1976) The echinoderms of southern Africa. London: British Museum (Natural History). 277 p.

Coleman N (2007). Sea stars: Echinoderms of the Asia/Indo-Pacific. Neville Coleman’s Underwater Geographic. Australia: Springwood. 136 p.

Thandar AS (1989) The sclerodactylid holothurians of southern Africa, with erection of one new subfamily and two new genera (Echinodermata: Holothuroidea). S Afr J Zool 24:290–304.

Thandar AS (1990) The phyllophond holothurians of southern Africa with the erection of a new genus. S Afr J Zool 25:207–223.

### Pycnogonida

Barnard KH (1954) South African Pycnogonida. Ann S Afr Mus 41:81–159.

### Crustacea

Barnard KH (1950) Descriptive catalogue of South African decapod Crustacea (crabs and shrimps). Ann S Afr Mus 38: 1–837.

Barnard KH (1950) Descriptive list of South African stomatopod Crustacea (Mantis shrimps) Ann S Afr Mus 38:838–864.

Berry PF (1971) The spiny lobsters (Palinuridae) of the East Coast of southern Africa: distribution and ecological notes. Investl Rep Ocean Res Inst S Afr 27:1–23.

Boden BP (1954) The euphausiid crustaceans of southern African waters. Trans Roy Soc S Afr 34:181–234.

Debelius H (1999) Crustacea guide of the world. Frankfurt: IKAN-Unterwasserarchiv. 321 p.

De Freitas AT (1985) The Penaeoidea of southern Africa 1. The study area and key to the southern African species. Investl Rep Ocean Res Inst S Afr 56:1–31.

Griffiths CL (1976) Guide to the benthic marine amphipods of southern Africa. Cape Town: South African Museum. 106 p.

Kensley B (1972) Shrimps and prawns of southern Africa. Cape Town: South African Museum. 65 p.

Kensley B (1978) Guide to the marine isopods of southern Africa. Cape Town: South African Museum. 173 p.

### Echiura and Sipuncula

Wesenberg-Lund E (1963) South African sipunculids and echiuroids from coastal waters. Vidensk Medd Dansk Naturh Foren 126:101–146.

### Polychaeta

Day JH (1967). A monograph on the Polychaeta of southern Africa. London: Trustees of the British Museum (Natural History). 878 p.

### Brachiopoda

Hiller N (1991). The southern African recent brachiopod fauna. In: Brachiopods through Time. McKinnon DI, Lee DE, Campbell JD, editors, Rotterdam: A.A. Balkema. pp. 439–445.

Jackson JW (1952) A revision of some South African Brachiopoda, with descriptions of new species. Ann S Afr Mus 41: 1-40.

### Mollusca

Debelius H (1998) Nudibranchs and sea snails Indo-Pacific field guide. Frankfurt: IKAN-Unterwasserarchiv. 321 p.

Gosliner T (1987) Nudibranchs of southern Africa - A guide to Opisthobranch molluscs of southern Africa. Monterey: Sea Challengers. 136 p.

Kaas P, Van Belle RA (1985–1991) Monograph of living chitons (in 4 volumes). E.J. Brill.

Kilburn R, Rippey E (1982) Sea shells of southern Africa. Johannesburg: Macmillan South Africa. 249 p.

Liltved WR (1989) Cowries and their relatives of southern Africa. A Study of the southern African Cypraeacean and Velutinacean gastropod fauna. Gordon Verhoef, Seacomber Publications. 208 p.

Norman M (2000) Cephalopods a world guide. Hackenheim: ConchBooks. 320 p.

Richards D (1981) Shells of southern Africa - A concise guide for collectors. Cape Town: Struik Publishers. 156 p.

Roeleveld MA (1972) A review of the Sepiidae (Cephalopoda) of southern Africa. Ann S Afr Mus 59: 193–313.

Steyn DW, Lussi M (2005) Offshore shells of southern Africa – A pictorial guide to more than 750 gastropods. Published by the authors.

Zsilavecz G (2007) Nudibranchs of the Cape Peninsula and False Bay. Cape Town: Southern Underwater Research Group Press. 104 p.

### Bryozoa

Florence WK, Hayward PJ, Gibbons MJ (2007). Taxonomy of shallow-water Bryozoa from the west coast of South Africa. Afr Nat Hist 3: 1–58.

### Cnidaria

Carlgren O (1938) South African Actinaria and Zoantharia. Kungl. Svensk. Vet.-Ahad. Hatidl. (Series 3) 17: 1–148.

Kramp PL (1961) Synopsis of the medusae of the world. J. Mar. Biol. Assoc. U.K. 40: 1–469.

Millard NAH (1975) Monograph on the Hydroida of southern Africa. Ann. S. Afr. Mus. 68: 1–513.

Pages S, Gili JM, Bouillin J (1992) Planktonic cnidarians of the Benguela Current. Scientia Marina 56 (Suppl. 1): 1–144.

Veron JEN (1986) Corals of Australia and the Indo-Pacific. North Ryde, Australia: Angus and Robertson. 644 p.

Williams GC (1990) The Pennatulacea of southern Africa (Coelenterata, Anthozoa). Ann S Afr Mus 99: 31–119.

Williams GC (1992) The Alcyonacea of southern Africa. Stoloniferous octocorals and soft corals (Coelenterata, Anthozoa). Ann S Afr Mus 100: 249–358.

Williams GC (1992) The Alcyonacea of southern Africa. Gorgonian octocorals (Coelenterata, Anthozoa). Ann S Afr Mus 101: 181–296.

Williams GC (1993) Coral reef octocorals. An illustrated guide to the soft corals, sea fans and sea pens inhabiting the coral reefs of northern Natal. Durban: Durban Natural Science Museum. 64 p.

### Porifera

Samaai T, Gibbons MJ (2005) Demospongiae taxonomy and biodiversity of the Benguela region on the west coast of South Africa. Afr Nat Hist 1:1–96.

### Angiospermae

Lubke RA, Van Wyk K (1998) Terrestrial plants and coastal vegetation. In: Field guide to the Eastern and Southern Cape coasts (2nd ed). Lubke R, De Moore I, editors. Cape Town: University of Cape Town Press. pp. 289–342.

Lubke RA, Van Wyk K (1998) Terrestrial plants and coastal vegetation. In: Field guide to the Eastern and Southern Cape coasts (2nd ed). Lubke R, De Moore I, editors. Cape Town: University of Cape Town Press. pp. 187–197.

Berjak P, Campbell GK, Huckett BI, Pammenter NW (1977) The mangroves of southern Africa. Durban: Natal Branch, Wildlife Society of Southern Africa. 73 p.

O'Callaghan M (1992) The ecology and identification of the southern African Salicomieae (Chenopodiaceae). S Afr J Bot 58: 430–439.

### Macroalgae

Stegenga H, Bolton J, Anderson R (1997) Seaweeds of the South African West Coast. Contrib. Bolus Herbarium 18: 1–655.

De Clerck O, Bolton JJ, Anderson RJ, Copperjans E (2002) Guide to the seaweeds of KwaZulu-Natal. Scripta Botanica Belgica 33: 1–294.
